# Modulated complexed stenosed region consequences under the electroosmotic stimulation

**DOI:** 10.1038/s41598-023-45210-3

**Published:** 2023-10-19

**Authors:** S. Ijaz, S. Shaheen, Iqra Shahzadi, Taseer Muhammad

**Affiliations:** 1 Department of Mathematics, Faculty of Sciences, Rawalpindi Women University, Rawalpindi, Pakistan; 2 Department of Mathematics, King Khalid University, 62529 Abha, Saudi Arabia

**Keywords:** Biophysics, Mathematics and computing, Nanoscience and technology

## Abstract

The present study analyzes the theoretical consequences of slip effects in a complex stenosed region. The flow of blood in a stenosed region is incorporated with hybrid nanofluid features which are being prepared with copper and copper oxide nanoparticles. The flow is also intensified by applying an electric field in the axial direction. The governing equations for the proposed paradigm are solved and the corresponding closed-form solutions are obtained for the cases of mild stenosis. Parameters such as Electro-osmotic, velocity slip and Helmholtz–Smoluchowski are specially focused in this study. The heat transfer, hemodynamic velocity, wall shear stress and resistance impedance for the flow are precisely determined. The various parameters that influence the physical characteristics of flow are plotted, and their effects are discussed in detail. The present model has the potential application in medical pumps for drug delivery systems.

## Introduction

Nanotechnology is concerned with the study of tiny objects and the configuration of matter. The nanoparticle’s structured dimension is one hundred nanometers. Nanotechnology can help to deliver medications more effectively to different parts of the body. This makes it easier for the medications to reach their target. This helps improve the efficiency of drug distribution in tissues and cells. Nanotechnology has made a significant impact on the field of science, with many potential applications being noted. By staring at a particular material and its structure, such substances are transformed into nanoparticles, in addition to copper nanoparticles, which can also determine new properties^1^. These latest technologies are very popular in medicine because of their benefits. Nanofluids made from two different types of nanoparticles are called hybrid nanofluids. They are different from regular nanofluids because they have a mix of the two types of nanoparticles. Hybrid nanoparticles are a special kind of compound that is made up of different parts and have both physical and chemical properties. They are often used in the production of anticancer pills. Several assessments have been made of hybrid nanofluids^2–10^. If the coronary arteries are narrow, this can cause a decrease in the amount of blood that can flow to the muscles of the heart, which can lead to death.

Arteriosclerosis is the main cause of the narrowing of arteries. Arteriosclerosis is a condition in which the arteries become thick and rigid, which can lead to myocardial infarction, coronary artery infections, strokes, cardiac arrests and angina^11–15^. When studying blood flow across narrowed arteries, it’s important to take into account nanoparticles. These tiny particles can play a significant role in controlling how well blood flows through the vessel. Abdel Salam et al.^16^, presented a model to examine the bloodstream of a non-Newtonian fluid using magneto electrodynamics using platelets, balls, and rod-like nanoparticles. Examination of nanoparticles in the bloodstream through permeable blocked arteries have been performed by Ellahi et al.^17^. They noticed that the height of the stenosis was directly proportional to the shear stress distribution. Nadeem et al.^18^ studied the effect of blood stream in the presence of a right-coronary stenosed artery. They used Carreau's model of fluid flow to simulate blood flow. The researchers found that hemodynamic characteristics are improved when stenosis is increased in the ratio.

When the fluid and the wall of the channel interact, a charge is naturally generated on the surface. Alternatively, the electrodes are used to generate the charge. The water flowing over this charged surface is called Electro-osmosis. This material is used in many different ways in engineering and medical fields. Bio-microfluidics frameworks use electroosmosis to analyze fluids. Electroosmosis is an important tool for flow detection in various applications. This electroosmotic transfer process is based on a method whereby an interface charger, known as the Stern layer, can attract oppositely charged particles from the electrolyte system. The Electric Double Layer (EDL) is a layer of charged particles that form on the charged sheet surface that is linked to an outer diffuse surface. When an electrical field is applied to move particles in an electric diffuser (EDL), they move in a way that creates a fluid displacement, known as Electroosmotic Flow. A large number of examinations were carried out in the references^19–23^.

Most of the flow of Newtonian and non-Newtonian fluids is studied considering the no-slip condition, while few efforts are devoted to the study of fluids under the impact of slip conditions at the boundary. Neill et al. research is valuable. Bassett and other data^24,25^ show that slip occurs at a solid boundary. For most problems, the roughness of the boundary surface can be important on a microscale. If the boundaries are smooth, the normal non-slip boundary conditions are not maintained and fluid slippage can occur on solid surfaces. Navier et al.^26^ suggested a boundary condition in which the velocity of the fluid near the solid boundary is related to the stress acting on that point. Experimental studies of Newtonian fluids in the presence of slip boundaries have been reviewed by Neto et al.^27^.

In light of this, various flow problems have been explored by numerous researchers taking the effects of slip conditions into account. The Brinkman number is often used to describe the impact of viscous dissipation. This number is used to characterize the rate at which a viscous fluid dissipates its energy. For microdevices with gas flow, the viscous dissipation effect is important, even at low velocities^28^. Viscous dissipation affects heat transfer in the flow of fluid through the channel and has been examined in both experimental and theoretical work. Due to its wide application, many authors have focused their attention to examine the flow of fluid under the impact of heat transfer. For example, Ramesh^29^ studied the impact of viscous dissipation and joule heating on the Poiseuille flows of a Jeffrey fluid with slip boundary conditions. Sheikholeslami^30^ conducted extensive studies of the influence of a magnetic field on the flow of a CuO-water nanofluid and heat transfer in a closed space heated from down.

This research is aimed at investigating how electroosmotic forces and viscous dissipation affect a diseased artery with stenosis, and how this affects heat transfer. Since nanoparticles are essential for improving delivery efficiency in vascular flow, their physicochemical properties are taken into account in this examination. This appears to be the first time such a study has been conducted. For cases of moderate stenosis, the task has been simplified and exact solutions for temperature, hemodynamic velocity, wall shear stress, and flow resistance impedance have been obtained. Several important flow phenomena have been discovered when examining graphs of flow parameters and streamlines. Lastly, the essential findings of the outcomes are concise at the end.

## Mathematical formulation

**Figure 1:**
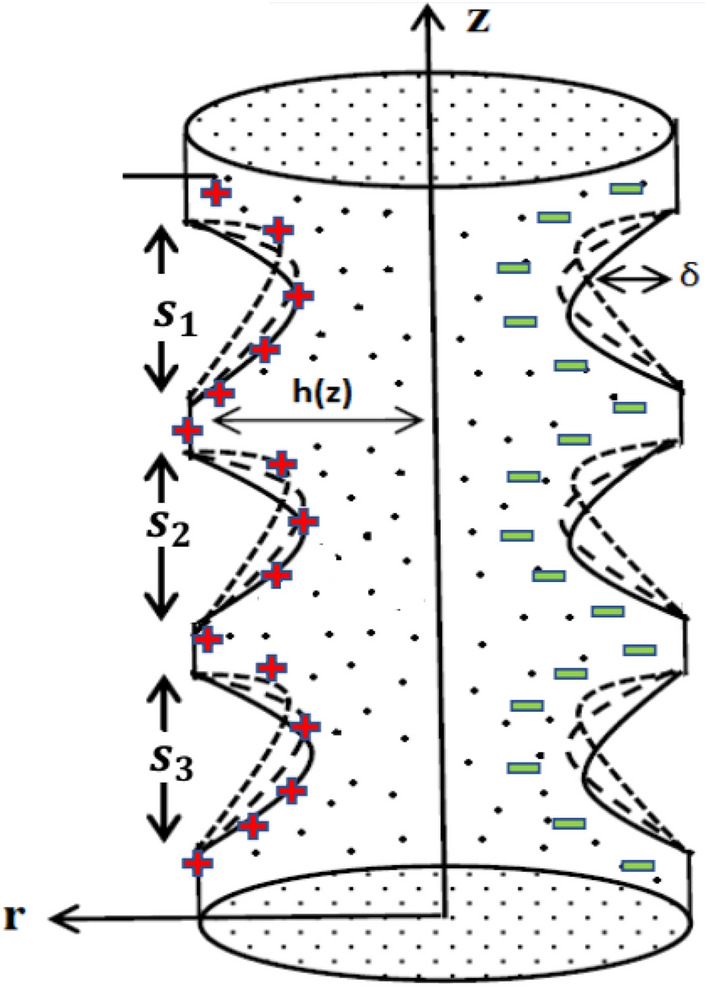
Geometry of catheter anisotropic region.

The above Fig. 1, we have some representations to describe the blood flow across the diseased artery such as, $\left( {\vec{r}, \vec{z}} \right)$ represents cylindrical coordinates*,*$\left( {\vec{u}, \vec{w}} \right)$ as velocities components along radial and axial direction,$z_{d}$ as axial displacement,$R_{1}$ for Non-stenotic radius of outer tube, $s_{l}$ used for stenosis length at $l = 1,2,3$ and stenosis location is represented by $d_{l}$ at $l = 1,2,3.$ Geometry, describing the blood flow across an artery with multiple stenosis at outer wall is considered as

1 $$\vec{h}{ }\left( {\text{z}} \right) = \left\{ {\begin{array}{*{20}l} {R_{1} \left[ {1{ } - K\left\{ {s_{l}^{n - 1} \left( {\vec{z} - d_{l} } \right) - \left( {\vec{z} - d_{l} } \right)^{n} } \right\}} \right], } \hfill & {d_{l} \le \vec{z} \le d_{l} + s_{l} } \hfill \\ {CR_{1} , } \hfill & {otherwise.} \hfill \\ \end{array} } \right.$$

In above, we have

2 $$K = \frac{{\delta_{l}^{*} }}{{R_{1} s_{l}^{n} }} \frac{{n^{{{\raise0.7ex\hbox{$n$} \!\mathord{\left/ {\vphantom {n {n - 1}}}\right.\kern-0pt} \!\lower0.7ex\hbox{${n - 1}$}}}} }}{n - 1}$$

where $\delta_{l}^{*}$ represents the utmost height attained by stenosis for $n = 2$ the case is for symmetric and for $n > 2$ the case is considered for non-symmetric stenosis. Also*,* $\vec{h}{ }\left( {\text{z}} \right){ }$ represents exterior boundary, i.e. (wall having multiple stenosis) is in their dimensional form.

## Mathematically governed flow problem

The experimental and theoretical analysis of two-dimensional viscous fluid model of diseased artery with electric field and homogenous mixture of hybrid nanofluid with blood as base fluid governs the following mathematical equations, which are in dimensional form is given as^11^

3 $$\frac{1}{{\vec{r}}}\frac{{\partial \left( {\vec{r},\vec{u} } \right)}}{{\partial \vec{r}}} + \frac{{\partial \vec{w}}}{{\partial \vec{z}}} = 0$$

4 $${ }\rho_{{hnf{ }}} \left[ {\vec{u}\frac{{\partial \vec{w}}}{{\partial \vec{r}}} + { }\vec{w}\frac{{\partial \vec{w}}}{{\partial \vec{z}}}} \right] = { } - { }\frac{{\partial \vec{\rho }}}{{\partial \vec{z}}} + \mu_{{hnf{ }}} { }\left[ {\frac{{\partial^{2} \vec{w}}}{{\partial \vec{r}^{2} }} + { }\frac{1}{{\vec{r}}}\frac{{\partial \vec{w}}}{{\partial \vec{r}}} + { }\frac{{\partial^{2} \vec{w}}}{{\partial \vec{z}^{2} }}{ }} \right] + \rho_{{e{ }}} E_{{Z{ }}}$$

5 $$\begin{aligned} \rho_{hnf } C_{p } \left[ {\vec{u}\frac{{\partial \vec{T}}}{{\partial \vec{r}}} + \vec{w}\frac{{\partial \vec{T}}}{{\partial \vec{z}}}} \right] & = k_{hnf } \left[ {\frac{{\partial^{2} \vec{T}}}{{\partial \vec{r}^{2} }} + \frac{1}{{\vec{r}}}\frac{{\partial \vec{T}}}{{\partial \vec{r}}} + \frac{{\partial^{2} \vec{T}}}{{\partial \vec{z}^{2} }} } \right] \\ & \quad + \mu_{hnf } \left[ {2\left\{ {\left( {\frac{{\partial \vec{u}}}{{\partial \vec{r}}}} \right)^{2} + \left( {\frac{{\partial \vec{w}}}{{\partial \vec{z}}}} \right)^{2} + \left( {\frac{{\vec{u}}}{{\vec{r}}}} \right)^{2} } \right\} + \left( {\frac{{\partial \vec{w}}}{{\partial \vec{r}}} + \frac{{\partial \vec{u}}}{{\partial \vec{z}}}} \right)^{2} } \right] + \theta_{0 } \\ \end{aligned}$$

where in above $\rho_{hnf } ,$ $k_{hnf } {\text{and}}$ $\mu_{hnf }$ respectively the attributes of hybrid nanofluid particles for copper (Cu) and copper-oxide (CuO) cases and their mathematical form are listed as below

6 $$\rho_{hnf } = \rho_{f } \left( {1 - \emptyset_{2}^{*} { }} \right){ }\left( {1 - \emptyset_{1}^{*} + { }\emptyset_{1}^{*} { }\frac{{\rho_{1} }}{{\rho_{f } }} { }} \right){ } + \emptyset_{2}^{*} \rho_{2 }$$

To approximate the viscosity of hybrid nanofluid, Brinkman’s viscosity model is used as^12^

7 $${\upmu }_{{\text{hnf }}} = \frac{{{\upmu }_{{\text{f }}} }}{{{ }\left( {1 - \emptyset_{2}^{*} { }} \right)^{2.5} { }\left( {1 - \emptyset_{1}^{*} { }} \right)^{2.5} }}.$$

Thermal conductivity of hybrid nanofluid mixture is approximated by Maxwell and Hamilton- crosser’s model given as^13^

8 $$\frac{{k_{hnf } }}{{k_{bf } }} = \frac{{k_{2 } + \left( {m_{1 } - 1} \right)k_{bf } - \left( {m_{1 } - 1} \right) \emptyset_{2}^{*} \left( {kb_{f } - k_{2 } } \right)}}{{k_{2 } + \left( {m_{1 } - 1} \right)k_{bf } + \emptyset_{1}^{*} \left( {kb_{f } - k_{2 } } \right)}},$$

9 $$\frac{{k_{bf } }}{{k_{f } }} = \frac{{k_{1 } + \left( {m_{1 } - 1} \right)k_{f } - { }\left( {m_{1 } - 1} \right){ }\emptyset_{1}^{*} { }\left( {k_{f } - k_{1 } } \right)}}{{k_{1 } + \left( {m_{1 } - 1} \right)k_{f } + { }\emptyset_{1}^{*} { }\left( {k_{f } - k_{1 } } \right)}}.$$

where

10 $$\emptyset = \emptyset_{1}^{*} + \emptyset_{2}^{*} .$$

In above $\emptyset_{1}^{*}$ and $\emptyset_{2}^{*}$ represents the volumetric fraction. Moreover, the thermophysical significance of hybrid nanofluid particle with base fluid are given as^13^

Poison–Boltzman equation for electric potential distribution in presence of the electric double layer is given as^14^

11 $$\left( {\nabla . \nabla } \right)\vec{\psi } = - \frac{{\rho_{e } }}{E},$$

where $\rho_{e }$ the case of binary fluid stands which for net charge density.and consists of two types of ions with equal and opposite charges, the net charge density is specified as,

12 $$\rho_{e } = {\text{e}}Z^{*} \left( {n^{ + } - n^{ - } } \right),$$

where $n^{ + } = n_{0 } e^{{\left( { + {\text{e}}z^{*} { }\frac{{\vec{\psi }}}{{K_{A} T^{*} }} } \right)}}$ and $n^{ - } = n_{0 } e^{{\left( { - {\text{e}}z^{*} { }\frac{{\vec{\psi }}}{{K_{A } T^{*} }} } \right)}}$ represents positive and negative charges in bulk concentration is the electric charge,$z^{*}$ is the valence of ions, $K_{A }$ is the Boltzmann constant, $T^{*}$ is the local absolute temperature of electrolyte solution and $n_{0 }$ is the average concentration of ions. By assuming the symmetricity of electrolytes, the net charge density described in equation can be computed as

13 $$\rho_{e } = - 2n_{0 } {\text{e}}Z^{*} \sinh \left( {{\text{e}}Z^{*} \frac{{\vec{\psi }}}{{K_{A } T^{*} }} } \right),$$

As $Sinh\left( {{\text{e}}Z^{*} { }\frac{{\vec{\psi }}}{{K_{A } T^{*} }} } \right)$ $\approx$ ${\text{e}}Z^{*} { }\frac{{\vec{\psi }}}{{K_{A } T^{*} }}$ is known as Debye–Huckel supposition, with this assumption the above expression may be computed as

14 $$\rho_{e } = - 2n_{0 } {\text{e}}Z^{*} \left( {{\text{e}}Z^{*} { }\frac{{\vec{\psi }}}{{K_{A} T^{*} }} } \right),$$

Utilizing non-dimensional parameters ${\text{r}} = \frac{{\vec{r}}}{{R_{1} }}$ and $\psi$ = $\frac{{\vec{\psi }}}{\xi }$ and putting the value of $\rho_{e }$ in Eq. (11), we get

15 $$\frac{1}{ r}\frac{\partial }{\partial r}{ }\left( { r\frac{{\partial \psi { }}}{\partial r } } \right) = m^{2} \psi .$$

By using $\frac{{\partial \psi { }}}{\partial r }$ = 0 at r = 0 and $\psi$ = 1 at r = $h$(z). Above Eq. (15) can be written as

16 $$\psi = \frac{{I_{0 } { }\left( {{\text{mr}}} \right)}}{{I_{0 } \left( {{\text{mh}}} \right)}}.$$

Dimensionless parameters are given as

17 $$\begin{gathered} {\text{r}} = \frac{{\vec{r}}}{{R_{1} }}, z = \frac{{\vec{z}}}{{s_{l} }},{\text{ u}} = \frac{{L\vec{u}}}{{u_{{o\delta_{l}^{*} }} }},{ } h\left( z \right) = \frac{{\vec{h}{ }\left( {\text{z}} \right)}}{{R_{1} }}, \delta_{l} = \frac{{\delta_{l}^{* } }}{{R_{1} }}, \hfill \\ p = \frac{{\vec{p}R_{1}^{2} }}{{u_{f} u_{o} s_{l} }} , h_{l} = \frac{{d_{l} }}{{s_{l} }}, \theta = \frac{{\vec{T} - \overrightarrow {{T_{0} }} }}{{\overrightarrow {{T_{0} }} }}, \theta_{0} = \left( {\frac{{T_{0} }}{{T_{1} - T_{0} }}} \right), \hfill \\ B_{r } = \frac{{u_{f} u_{o}^{2} }}{{k_{f } { }\left( {\overrightarrow {{T_{1} }} - \overrightarrow {{T_{0} }} } \right)}}, S_{{G_{o} }}^{*} = \frac{{k_{f} }}{{\overrightarrow {{T_{o} }}^{2} }}\left[ {\left( {\frac{{T_{1} - T_{0} }}{{R_{1} }}} \right)^{2} } \right], \psi = \frac{{\vec{\psi }}}{\xi }, {\text{U }} = \frac{{E\xi E_{Z } }}{{\mu_{hnf } u_{o} }}, \hfill \\ {\text{w}} = \frac{{\vec{w}}}{{u_{o} }},{ }m = R_{1} {\text{e}}Z^{*} \sqrt {\frac{{2n_{0 } }}{{K_{A } T^{*} {\text{E }}}}{ }} , \beta = \frac{{R^{2} }}{{k_{f } T_{0} }}\theta_{0 } \hfill \\ \end{gathered}$$

By substituting Eqs. (16) and (17) in Eqs. (3–5), we obtained the following system of equations and considering the assumption of mild stenosis $$\frac{{\delta_{l}^{* } }}{{R_{1} }} \ll 1$$ and $$\frac{{n^{{{\raise0.7ex\hbox{$1$} \!\mathord{\left/ {\vphantom {1 {n - 1}}}\right.\kern-0pt} \!\lower0.7ex\hbox{${n - 1}$}}}} R_{1} }}{{s_{l} { }}} \approx O\left( 1 \right)$$

18 $$\frac{\partial p}{{\partial r}} = 0,$$

19 $$\frac{\partial p}{{\partial z}} = A_{2} { }\left[ {\frac{{\partial^{2} w}}{{\partial r^{2} }} + \frac{1}{r} \frac{\partial w}{{\partial r}}} \right] + m^{2} U,$$

20 $$\frac{{\partial^{2} \theta }}{{\partial r^{2} }} + \frac{1}{r} + \frac{\partial \theta }{{\partial r}} + B_{r } A_{3 } \left( {\frac{\partial w}{{\partial r}}} \right)^{2} + A_{4 } \beta = 0$$

Constants are described as below

21 $$S_{{4{ }}} = { }\frac{{k_{{1{ }}} + \left( {m_{{1{ }}} - 1} \right)k_{{f{ }}} - { }\left( {m_{{1{ }}} - 1} \right){ }\emptyset_{1}^{*} { }\left( {k_{{f{ }}} - k_{{1{ }}} } \right)}}{{k_{{1{ }}} + \left( {m_{{1{ }}} - 1} \right)k_{{f{ }}} + { }\emptyset_{1}^{*} { }\left( {k_{{f{ }}} - k_{{1{ }}} } \right)}},$$

22 $$S_{5 } = \frac{{k_{2 } + \left( {m_{1} - 1} \right)k_{bf } - { }\left( {m_{1} - 1} \right){ }\emptyset_{2}^{*} { }\left( {k_{bf } - k_{2 } } \right)}}{{k_{2 } + \left( {m_{1 } - 1} \right)k_{bf } + { }\emptyset_{1}^{*} { }\left( {k_{bf} - k_{2 } } \right)}}\left( {S_{4 } } \right),$$

23 $$A_{2} = \left( {1 - \emptyset_{2}^{*} { }} \right)^{2.5} { }\left( {1 - \emptyset_{1}^{*} { }} \right)^{2.5} ,$$

24 $$A_{{3 = { }}} \left( {\frac{1}{{S_{5 } }}} \right)\left( {\frac{1}{{A_{2 } }}} \right),$$

25 $$A_{4 } = \frac{1}{{S_{5 } }}.$$

The relevant dimensionless boundary conditions are as^27–29^

26 $$\frac{\partial \theta }{{\partial r}} = 0{\text{ at r}} = 0{\text{ and }}\theta + \gamma \frac{\partial \theta }{{\partial r}} = 0 at r = h,$$

27 $$\frac{\partial w}{{\partial r}} = 0{\text{ at r}} = 0{\text{ and }}w + B_{2 } \frac{\partial w}{{\partial r}} = 0 {\text{ at}} r = h.$$

where

28 $$B_{2 = } \eta \frac{{\mu_{hnf } }}{{\mu_{f } }}.$$

By utilizing dimensionless parameters in Eq. (17), we get the corresponding dimensionless stenosis boundary conditions as

29 $$h\left( {\text{z}} \right) = \left\{ {\begin{array}{*{20}l} {1 - s_{l} \frac{{n^{{\frac{n}{n} - 1}} }}{n - 1}\{ \left( {z - h_{l} } \right) - (z - h_{l} )^{n} \} } \hfill & {whenever h_{l} \le z \le h_{l} + 1} \hfill \\ 1 \hfill & {otherwise. } \hfill \\ \end{array} } \right.$$

## Exact solution of mathematical model

Above evaluated system of non-dimensional equations from (18)–(20) are solved with associated non dimensional boundary conditions mentioned in Eqs. (26–27) with the help of computational software Mathematica. We get the following solutions.

### Velocity profile

By solving Eq. (19) with dimensionless boundary conditions mentioned in Eq. (26), we get the velocity profile as

30 $$w\left( r \right) = \frac{1}{4}A_{2} \frac{\partial p}{{\partial z}}\left( { - h\left( {2B_{2} + h} \right)r^{2} } \right) + A_{2} U + \frac{{A_{2} U\left( { - A_{2} I_{o } \left( {{\text{mr}}} \right) + B_{{2{ }}} m} \right)I_{1 } \left( {{\text{mh}}} \right)}}{{I_{o } \left( {{\text{mh}}} \right)}}$$

The evaluated velocity profile in Eq. (30) is used to fined rate of flow as

31 $${\text{F}} = \mathop \smallint \limits_{0}^{{\text{h}}} rw dr,$$

32 $$F = \frac{1}{16}hA_{2} \left( { - h\left( {4B_{2 } h\frac{\partial p}{{\partial z}} + h^{2} \frac{\partial p}{{\partial z}} - 8U} \right) + \frac{{8\left( { - 2 + B_{2 } hm^{2} } \right)UI_{1 } \left( {mh} \right)}}{{mI_{o } \left( {mh} \right)}}} \right)$$

The expression of pressure gradient is obtained from above Eq. (32) and defined as

33 $$\frac{dp}{{ dz}} = \frac{{F - A_{6 } }}{{A_{5} }},$$

where

34 $$A_{5} = \frac{1}{16}A_{2} h\left( { - h\left( {4B_{2} h + h^{2} } \right)} \right),$$

35 $$A_{6} = \frac{1}{16}A_{2} h\left( {8Uh + \frac{{8\left( { - 2 + B_{2} hm^{2} } \right)UI_{1 } { }\left( {{\text{mh}}} \right)}}{{ mI_{0 } \left( {{\text{mh}}} \right)}} } \right)$$

The expression for wall shear stress is defined as

36 $$S_{rz} = - \frac{1}{{A_{2} }}\left. { \left( {\frac{\partial w}{{ \partial r}} } \right) } \right|_{r = h} .$$

Using Eq. (30) in above equation we get

37 $$S_{rz} = \left[ { - \frac{h}{2}{ }\left( { \frac{\partial p}{{\partial z}} } \right) + \frac{{mUI_{1 } { }\left( {{\text{mh}}} \right)}}{{mI_{0 } \left( {{\text{mh}}} \right)_{ } { }}} } \right].$$

### Temperature profile

The temperature profile is evaluated by considering Eq. (20) with dimensionless boundary conditions given in Eq. (27) as

38 $$\begin{gathered} \theta = \frac{1}{64}((16A_{4} \beta \left( {h^{2} - r^{2} + 2h\gamma } \right) + \frac{1}{{m^{2} }} \left( {A_{2}^{2} A_{3} B_{r } (h^{4} m^{2} \left( { \frac{\partial p}{{\partial z}} } \right)^{2} - m^{2} \left( { \frac{\partial p}{{\partial z}} } \right)^{2} } \right. \hfill \\ \left. {\left( { \frac{\partial p}{{\partial z}} } \right)U + 32m^{2} U^{2} - 32h^{2} m^{4} U^{2} + 4hm^{2} \left( {h^{2} \left( { \frac{\partial p}{{\partial z}} } \right)^{2} - 8U\left( {2\left( { \frac{\partial p}{{\partial z}} } \right) + m^{2} U} \right)} \right)\gamma } \right) \hfill \\ + \frac{1}{{m^{2} I_{0} \left( {hm} \right)^{2} }}32A_{2}^{2} A_{3} B_{r } U(I_{0} \left( {hm} \right)( - 4\left( { \frac{\partial p}{{\partial z}} } \right)I_{0} \left( {mr} \right) + m( - 2h\left( { \frac{\partial p}{{\partial z}} } \right) + hm^{2} U \hfill \\ + 4\left( { \frac{\partial p}{{\partial z}} } \right)\gamma + 2m^{2} U\gamma )\left( {I_{1} \left( {hm} \right) + 2m\left( { \frac{\partial p}{{\partial z}} } \right)rI_{1} \left( {mr} \right)} \right) + m^{2} U(((\left( { - 1 + m^{2} r^{2} } \right) \hfill \\ I_{0} \left( {mr} \right)^{2} - mrI_{0} \left( {mr} \right)I_{1} \left( {mr} \right) + m^{2} \left( {h\left( {h + \gamma } \right)I_{1} \left( {hm} \right)^{2} r^{2} I_{1} \left( {mr} \right)^{2} } \right))). \hfill \\ \end{gathered}$$

## Graphical configuration and findings

This section facilitates to discuss the exact results evaluated for velocity, pressure gradient and wall shear stress of flow through stenosed artery graphically by comparison of Cu-CuO/blood, Cu-blood and pure blood in a channel with electroosmosis effect. This analysis comprises experimental results of Cu-CuO/blood, Cu-blood nano particles cases which are listed in Tables 1 and 2. The current phenomena are discussed by using Helmholtz-Smoluchowski velocity approximately equal to the 2 cm^−1^ ionic meditation ranging from 1m-1Mm. Electric field of strength up to 1kV cm^−1^ is applied by knowing about the relative permittivity of base fluid^22,23^. Graphical configurations are plotted by considering laminar flow with hybrid nanofluid, and pure blood as base fluid with electroosmotic effect with different parameters of interest. The solutions evaluated above are plotted against the radial coordinate with enhanced values of dimensionless parameters of interest.

**Table 1:** Different shape features of nanoparticles^9^.

Geometrical appearance			
Shape	Bricks	Cylinder	Platelets
Shape factor	3.7	4.9	5.7

**Table 2:** Thermophysical significances of Cu, CuO and base fluid blood^9^.

Physical Characteristics	Basefluid (blood)	Nanoparticle (Cu)	Nanoparticle (CuO)
		
$\ddot{c}_{p} ({\text{J/kgK}})$	4179	385	540
$\ddot{\rho }\left( {\frac{{{\text{kg}}}}{{{\text{m}}^{3} }}} \right)$	1063	8933	6320
$\sigma \left( {I{/}\Omega {\text{m}}} \right)$	0.05	59.6 × 10^6^	2.7 × 10^−8^
$\ddot{k}_{f} ({\text{W/mK}})$	0.492	400	76.5

### Velocity profile

**Figure 2:**
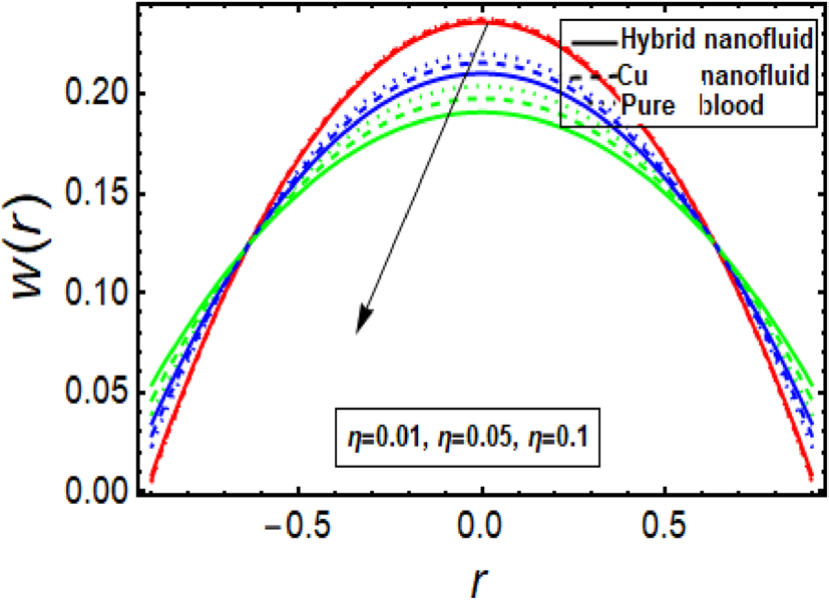
Velocity profile in a channel for velocity slip parameter.

**Figure 3:**
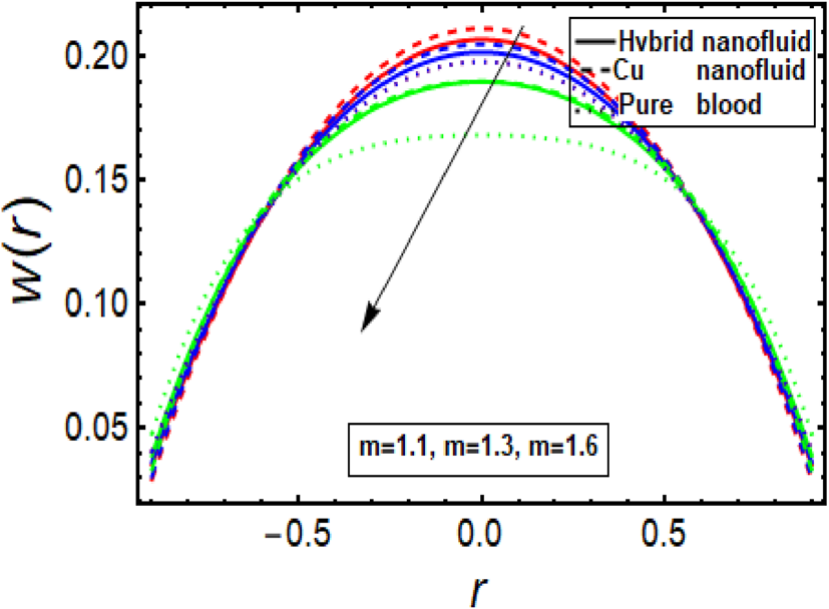
Velocity profile in a channel for parameter m.

**Figure 4:**
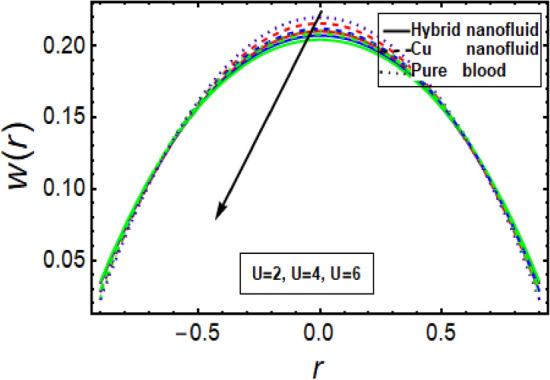
Velocity profile for Helmholtz–Smoluchowski (HS) velocity parameter.

**Figure 5:**
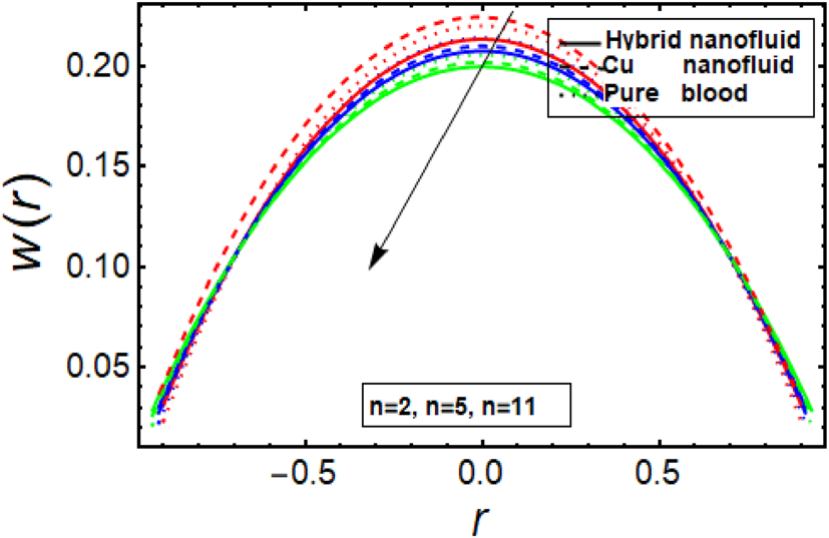
Velocity profile in a channel for multiple stenosis shape parameter.

The velocity profile depicts flow behavior in stenosed artery. The velocity of a viscous fluid flow in a channel with a steonsis at the outer walls is discussed using a graphical configuration. Figures 2, 3, 4 and 5 are planned to discuss the velocity significance for Cu-CuO/blood, Cu-blood nanoparticles and pure blood cases for different enhanced values of parameter η (velocity slip) *m*, *n*(stenosis shape) and *U*. In Fig. 2, We find that increasing η results in an increase in fluid velocity near the walls with stenosis, but a decrease in fluid velocity in the middle of the channel. To find out the visible effect of the parameter m on the fluid velocity, Fig. 3 is sketched and noted that by enhancing its value, the corresponding result is a deceleration at the center of the flow channel. Graphical result displayed in Fig. 4 against Helmholtz–Smoluchowski (HS) velocity parameter which shows minimum flow velocity output at the center of fluid flow channel but opposite behavior is noted near the walls having multiple stenosis. Velocity profile for symmetric and non-symmetric shape parameter *n* is designed in Fig. 5, it shows parabolic trajectory for both symmetric and non-symmetric multiple stenosis and profile of velocity decreases in the center of the channel more prominently as compared to the walls of the artery. It has been observed that the velocity of hybrid nanofluid near the arterial wall is much higher than the nanofluid and the base fluid.

### Temperature significance

**Figure 6:**
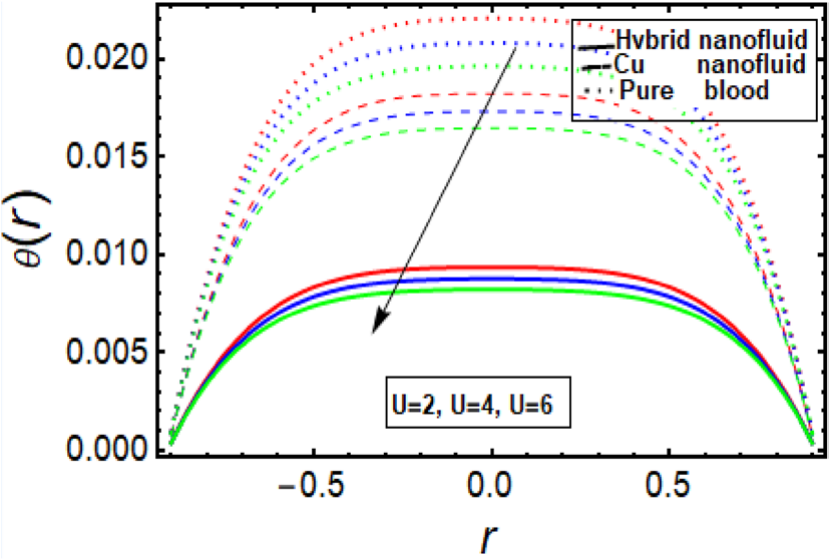
Temperature profile for Helmholtz–Smoluchowski (HS) velocity parameter.

**Figure 7:**
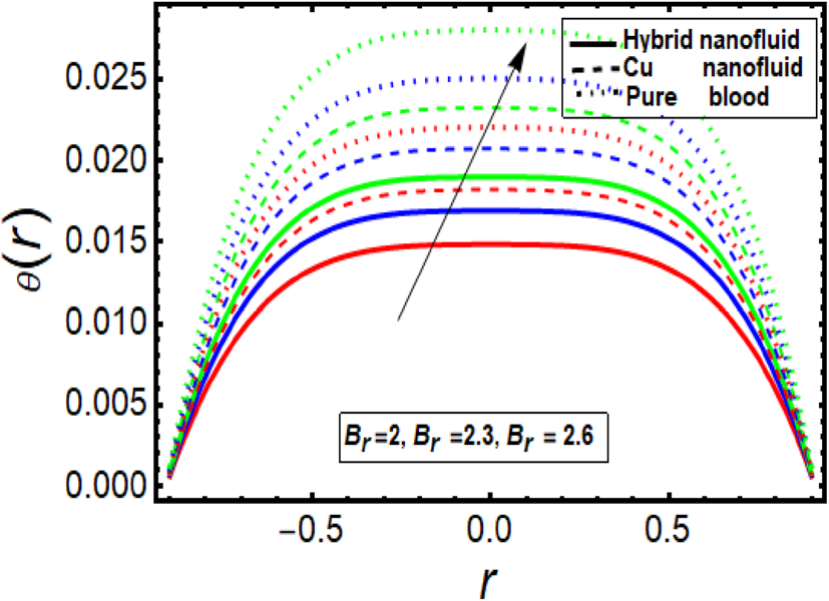
Temperature profile in a channel for parameter Brinkman number.

**Figure 8:**
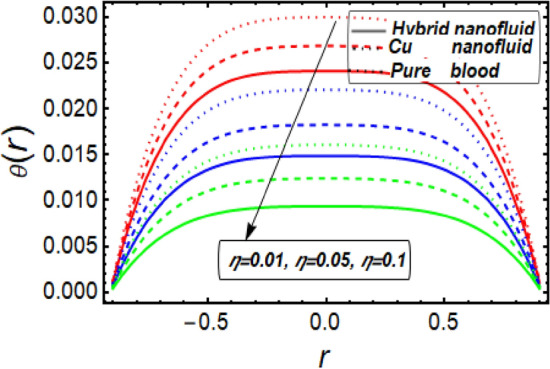
Temperature profile in a channel for velocity slip parameter.

**Figure 9:**
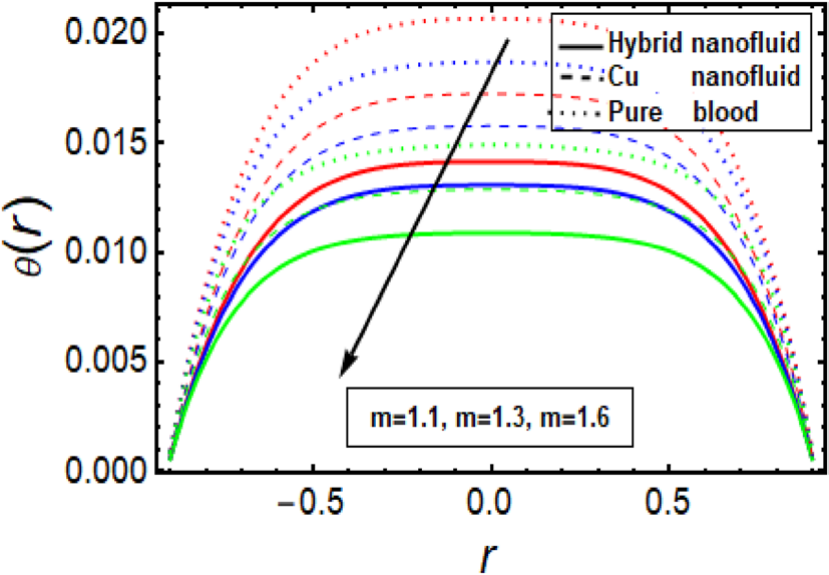
Temperature profile in a channel for parameter m.

**Figure 10:**
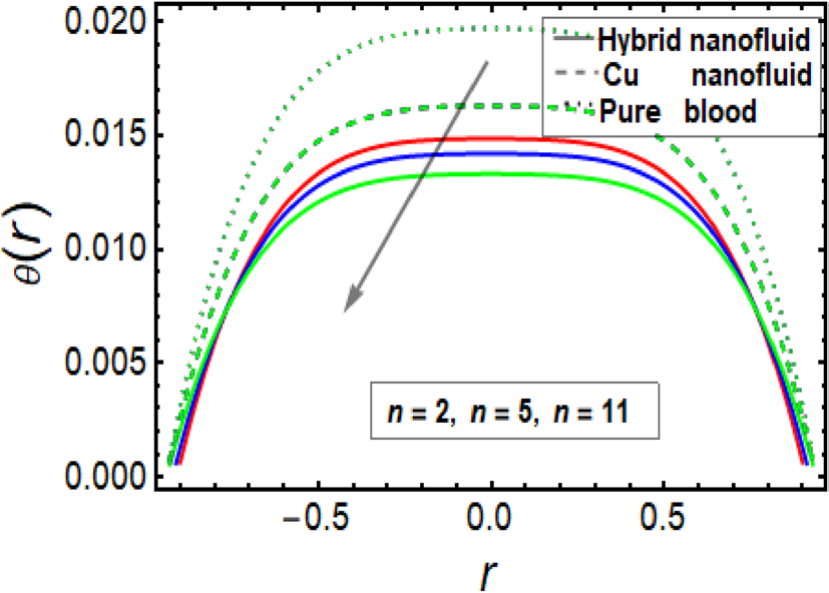
Temperature profile in a channel for multiple stenosis parameters.

This section facilitates to discuss the heat transfer significance through diseased artery having multiple stenosis at outer walls with applied electric field phenomena. The heat transfer rate is discussed through significant physical parameters of interest used in the present fluid flow model. In Fig. 6 it is noted that there is decline in temperature by enhancing values of Helmholtz–Smoluchowski (HS) velocity parameter. It happens due to assisting the electric field which will arise fluid velocity and hence minimum heat is generated within the fluid flow channel. To analyze the influence of Brinkman number (*B*_*r*_) on temperature profile, Fig. 7 is designed and It was noted that increasing the values of this parameter led to an increase in the temperature profile. It is perceived from Fig. 8 that velocity sip parameter declines the temperature profile by enhancing values of parameter. Influence of parameter *m* on temperature profile is found in Fig. 9. It is observed from this phenomenon that an increase in this parameter appears to lead to a decline in the temperature profile. Figure 10 is designed to discuss the temperature profile for symmetric and non-symmetric stenosis which shows decline in temperature profile for both the cases. In all these graphs, it is found that temperature is higher at the middle of channel as compared to the stenosed walls having electroosmosis effect. Thus, the rate of temperature flow can be reduced by the application of electric field. These graphs show that the temperature profile of a hybrid nanofluid (Cu–Cu/blood) is less than the temperature profile of a nanofluid (Cu-blood) and much more than a base fluid. Thus, the temperature of the fluid flow through the diseased artery with multiple stenosis can be controlled by injecting the nanoparticles into the blood.

### Shear stress graph

**Figure 11:**
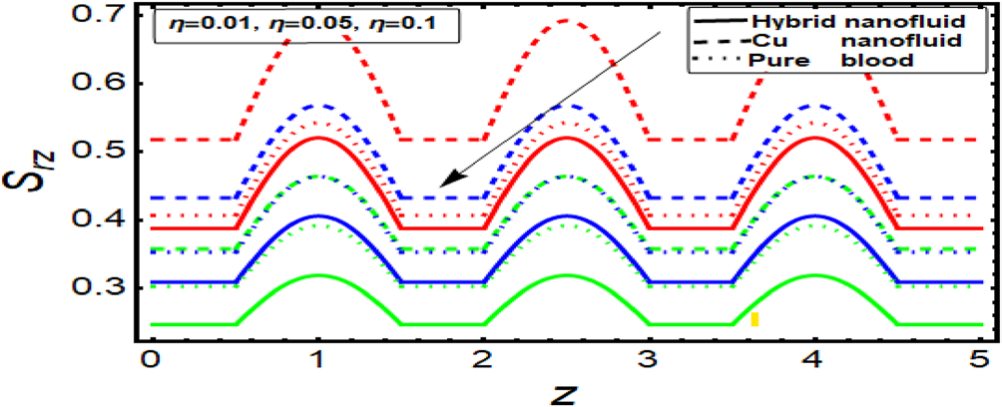
Shear stress on stenotic walls for velocity slip parameter.

**Figure 12:**
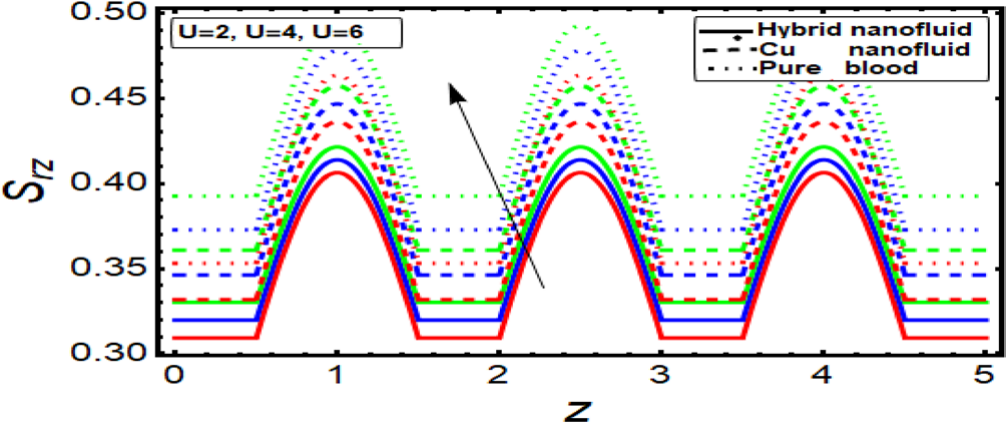
Shear stress for Helmholtz–Smoluchowski (HS) velocity parameter.

**Figure 13:**
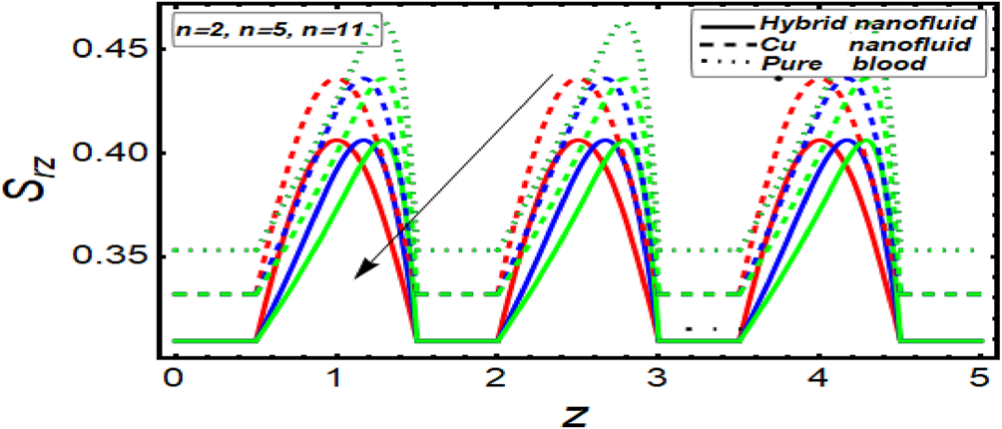
Shear stress for parameter.

**Figure 14:**
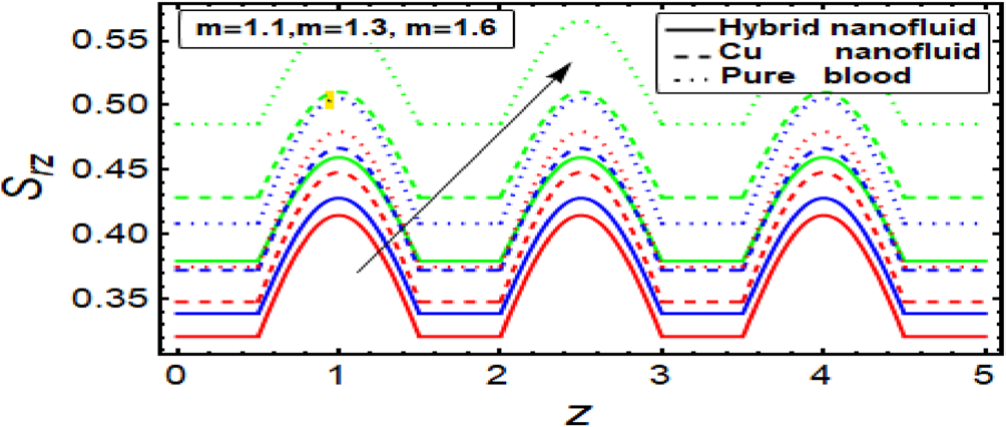
Shear stress for multiple stenosis shape parameter.

To analyze the behavior of stress on the stenotic wall, shear stress graphs are planned and displayed in Fig. 11, 12, 13 and 14 for different significant dynamical and thermodynamically feature based parameters of interest. In Fig. 11 it is noted that there is decline in wall shear stress by enhancing values of velocity slip parameter. Figures 12 and 13 are designed against enhancing parameters Helmholtz–Smoluchowski (HS) velocity parameter and *m*. But opposite behavior is found for these parameters. All these graphs depict that the shear stress profile of hybrid nanofluid (CuO–Cu/blood) is less than the nano fluid (Cu-blood) and much more than base fluid.

### Flow configuration through streamlines

**Figure 15:**
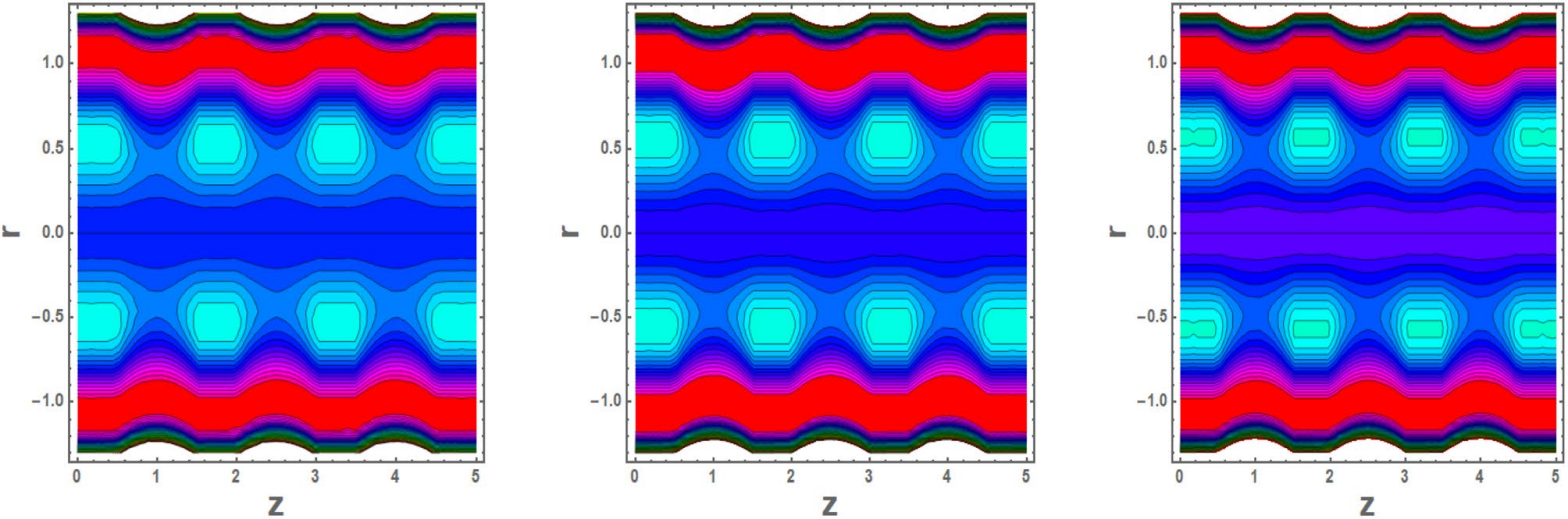
Blood flow pattern in multiple stenosed region versus different values (15a) m = 2.2 (15b) m = 2.4 (15c).

**Figure 16:**
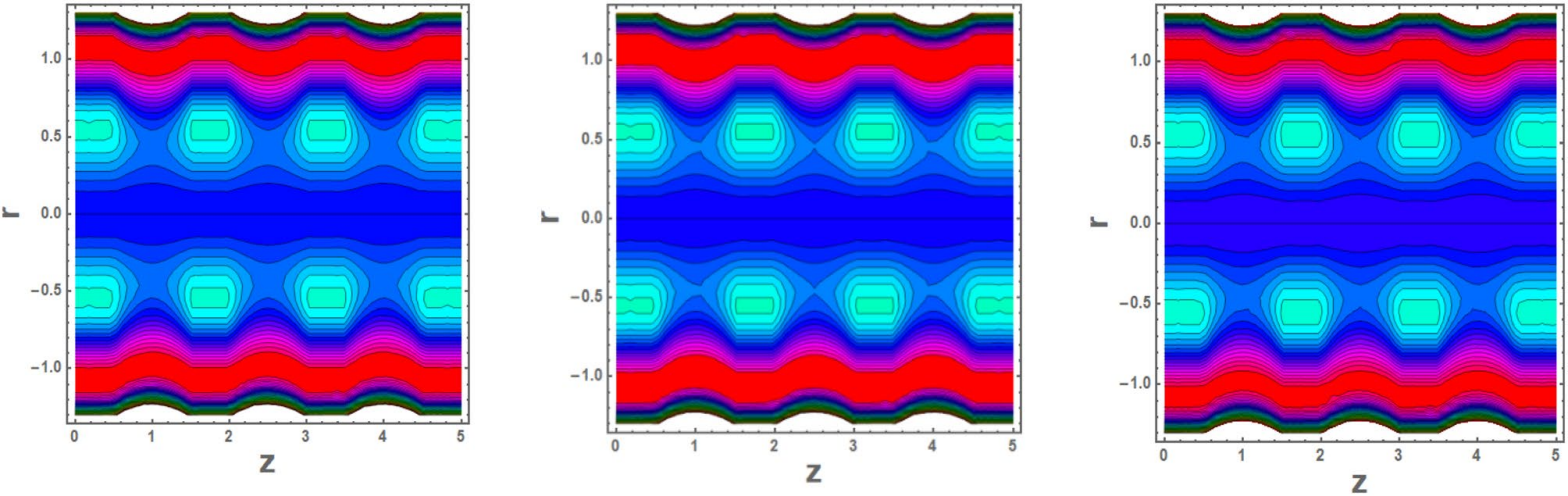
Blood flow pattern in in multiple stenosed region versus different values of for (16a) *U* = 2.2 (16b), *U* = 2.6 (16c) *U* = 2.6.

Trapping is an interesting occurrence in which a bolus is transported at the speed rate of waves. Figures 15a–c and 16a–c are plotted to see the significance of Helmholtz–Smoluchowski (HS) velocity and parameter *m* on flow pattern. It is concluded from these configurations 15a, 15b, 15c, 16a, 16b, 16c that the random motion of fluid defining boluses occurs in a channel under the electroosmosis impacts. These illustrations portray that the Helmholtz–Smoluchowski (HS) velocity parameter plays a key role in accelerating flow more rapidly in presence of nanofluid characteristics.

## Conclusions

Theoretical model of blood flow across a diseased artery having multiple stenosis at outer walls is analyzed and the flow is refined by using a blood model with electric field effects. Exact solutions of mathematical model are plotted to conclude significant outputs of considered model. Key significant outcomes of the present investigation are summarized as,

- Enhancement in heat transfer rate is noted for Brinkman number.
- Upon increasing velocity slip parameter fluid particles moves faster near the walls but opposite behavior in the middle of channel.
- Shear stress through stenotic artery increases with enhancement in Helmholtz–Smoluchowski (HS) velocity parameter.
- Trapping bolus in stream flow pattern expands by enhancing Helmholtz–Smoluchowski (HS) velocity parameter.

## Data Availability

The authors states that all the data is provided in the paper no hidden file or data is essential however if journal required any further data from us, we will provide and the corresponding author is responsible to provide to the journal.
